# The chromosome-level genome of *Gypsophila paniculata* reveals the molecular mechanism of floral development and ethylene insensitivity

**DOI:** 10.1093/hr/uhac176

**Published:** 2022-08-24

**Authors:** Fan Li, Yuan Gao, Chunlian Jin, Xiaohui Wen, Huaiting Geng, Ying Cheng, Haoyue Qu, Xing Liu, Shan Feng, Fan Zhang, Jiwei Ruan, Chunmei Yang, Liangsheng Zhang, Jihua Wang

**Affiliations:** Floriculture Research Institute, Yunnan Academy of Agricultural Sciences, National Engineering Research Center for Ornamental Horticulture, Key Laboratory for Flower Breeding of Yunnan Province, 650200, Kunming, China; Shenzhen Branch, Guangdong Laboratory of Lingnan Modern Agriculture, Genome Analysis Laboratory of the Ministry of Agriculture and Rural Affairs, Agricultural Genomics Institute at Shenzhen, Chinese Academy of Agricultural Sciences, 518120, Shenzhen, China; Floriculture Research Institute, Yunnan Academy of Agricultural Sciences, National Engineering Research Center for Ornamental Horticulture, Key Laboratory for Flower Breeding of Yunnan Province, 650200, Kunming, China; College of Agriculture and Biotechnology, Zhejiang University, 310030, Hangzhou, China; Floriculture Research Institute, Yunnan Academy of Agricultural Sciences, National Engineering Research Center for Ornamental Horticulture, Key Laboratory for Flower Breeding of Yunnan Province, 650200, Kunming, China; School of Agriculture, Yunnan University, 650504, Kunming, China; Floriculture Research Institute, Yunnan Academy of Agricultural Sciences, National Engineering Research Center for Ornamental Horticulture, Key Laboratory for Flower Breeding of Yunnan Province, 650200, Kunming, China; School of Agriculture, Yunnan University, 650504, Kunming, China; Fujian Provincial Key Laboratory of Haixia Applied Plant Systems Biology, Fujian Agriculture and Forestry University, 350002, Fuzhou, China; Fujian Provincial Key Laboratory of Haixia Applied Plant Systems Biology, Fujian Agriculture and Forestry University, 350002, Fuzhou, China; Key Laboratory of Horticultural Plant Biology, Ministry of Education, College of Horticulture and Forestry Sciences, Huazhong Agricultural University, Wuhan 430070, Hubei Province, China; National R&D Center for Citrus Postharvest Technology, College of Horticulture and Forestry Sciences, Huazhong Agricultural University, Wuhan 430070, Hubei Province, China; Key Laboratory of Horticultural Plant Biology, Ministry of Education, College of Horticulture and Forestry Sciences, Huazhong Agricultural University, Wuhan 430070, Hubei Province, China; National R&D Center for Citrus Postharvest Technology, College of Horticulture and Forestry Sciences, Huazhong Agricultural University, Wuhan 430070, Hubei Province, China; Hubei Hongshan Laboratory, Wuhan 430070, China; Floriculture Research Institute, Yunnan Academy of Agricultural Sciences, National Engineering Research Center for Ornamental Horticulture, Key Laboratory for Flower Breeding of Yunnan Province, 650200, Kunming, China; Floriculture Research Institute, Yunnan Academy of Agricultural Sciences, National Engineering Research Center for Ornamental Horticulture, Key Laboratory for Flower Breeding of Yunnan Province, 650200, Kunming, China; College of Agriculture and Biotechnology, Zhejiang University, 310030, Hangzhou, China; Floriculture Research Institute, Yunnan Academy of Agricultural Sciences, National Engineering Research Center for Ornamental Horticulture, Key Laboratory for Flower Breeding of Yunnan Province, 650200, Kunming, China

## Abstract

*Gypsophila paniculata*, belonging to the Caryophyllaceae of the Caryophyllales, is one of the most famous worldwide cut flowers. It is commonly used as dried flowers, whereas the underlying mechanism of flower senescence has not yet been addressed. Here, we present a chromosome-scale genome assembly for *G. paniculata* with a total size of 749.58 Mb. Whole-genome duplication signatures unveil two major duplication events in its evolutionary history: an ancient one occurring before the divergence of Caryophyllaceae and a more recent one shared with *Dianthus caryophyllus*. The integrative analyses combining genomic and transcriptomic data reveal the mechanisms regulating floral development and ethylene response of *G. paniculata*. The reduction of *AGAMOUS* expression probably caused by sequence polymorphism and the mutation in miR172 binding site of *PETALOSA* are associated with the double flower formation in *G. paniculata*. The low expression of *ETHYLENE RESPONSE SENSOR* (*ERS*) and the reduction of downstream *ETHYLENE RESPONSE FACTOR* (*ERF*) gene copy number collectively lead to the ethylene insensitivity of *G. paniculata*, affecting flower senescence and making it capable of making dried flowers. This study provides a cornerstone for understanding the underlying principles governing floral development and flower senescence, which could accelerate the molecular breeding of the Caryophyllaceae species.

## Introduction

The genus *Gypsophila* belongs to the Caryophyllaceae family, and comprises ~150 species of the annual, biennial, and perennial plants which mainly originated from temperate Asia and Europe [1]. Among them, *G. paniculata* (2n = 34) is a perennial herbaceous shrub, the only species used as cut flowers in the genus *Gypsophila* [2]. With the clouds of tiny white or pink flowers covering the bunches of branching stems after blooming, *G. paniculata* is commonly used as fresh or dried filler in flower arrangements and bouquets. Due to its ornamental value, *G. paniculata* is listed in the top ten best-selling cut flowers in the global floricultural market [3]. In addition, the plants of *G. paniculata* contain various bioactive compounds with potential medicinal values, such as flavonoids, triterpene saponins, sterols, and volatiles, which increase the utilization and economic value of this ornamental plant [4].

Flower type is one of the most critical ornamental traits and thetop interest of many floricultural species (e.g. rose, carnation, and lisianthus). Among those diverse flower types, the double flower is considered to be the key and precious one. Thereby, breeding efforts have been invested in the creation and improvement of desirable double flower varieties [5]. The same holds for the breeding of *G. paniculata*, and several commercial varieties were released, such as ‘Million Stars’ and ‘Huixing 1’ [6]. However, the main breeding methods of this species are confined within conventional hybridization and subsequent phenotypic selection for specific traits, which become inoperative in the breeding of the double flower varieties due to their almost infertile reproductivesystem [7]. As a consequence, the variations existing among the current commercial cultivars are limited [8].

In the past decades, the genetic and molecular networks regulating flower development have been investigated in roses, carnations, and gerberas [9–11]. An ABCE model has been raised in the model plant *Arabidopsis* which also fits other species [12, 13]. The model indicates that the sepal formation is controlled by the A-class genes, which also function in the petal formation, together with the B-class genes. C-class gene specifies the stamen development with B-class genes and determines the carpel fate while alone. And the E-class genes function throughout the whole flower identity. In addition, the A- and C-class genes are antagonistic in expression patterns*.* The loss-of-function of *AGAMOUS* (*AG*) results in the elevated expression of A-class genes in the stamens, turning them into petals [14]. Similarly, the over-accumulation of *APETALA2*/*PETALOSA* (*AP2*/*PET*) due to the defects of miR172 binding reduces the *AG* expression in the stamens, leading to the double flower phenotype likewise [9, 10]. Although this model has been verified in many ornaments, it remains unclear whether it is true in *G. paniculata* [15].

Flowers that remain open and colored long after senescence initiation are normally used as dried flowers, such as species in *Limonium* and *Gypsophila* [16]. This phenomenon may relate to ethylene regulation, as ethylene functions critically in flower senescence and petal abscission [17, 18]. In addition to ethylene, phytohormones such as abscisic acid (ABA), cytokinin (CTK), gibberellins (GAs), auxin, and jasmonic acid (JA) also influence petal senescence in a synergistic or antagonistic way. The crosstalk between different phytohormones in petal senescence and the genes involved in relative biological processes are well reviewed [19]. Moreover, transcription factors like *RhHB1*, *RhMYB108*, *RhWRKY33*, and *DcWRKY75* affect petal senescence through positive or negative regulation of phytohormone synthesis and signal transduction [20–23]. Previous studies showed that the flowers of *G. paniculata* were highly sensitive to ethylene, and the endogenous ethylene content instantaneously increased in flower initiating senescent process [24, 25]. However, the flower decay or petal abscission occurred rarely during flower senescence, even though the petals became translucent, rolled, wilted, and desiccated. Abscission of petals exists in many species, and this process is highly sensitive to ethylene but not associated with petal senescence [26, 27]. To date, the knowledge of the molecular mechanisms underlying the relationship between ethylene response and petal senescence in *G. paniculata* flowers is limited.

In this study, a chromosome-level genome assembly of *G. paniculata* was presented. The genome was sequenced by a combination of long-read sequencing and Hi-C scaffolding technologies. In total, a 749.58 Mb genome was assembled and anchored to 17 pseudo-chromosomes. The molecular basis of double flower formation and regulation in *G. paniculata* was revealed through gene family and transcriptome analyses generated from diverse floral developmental stages, together with a forward genetic ethyl methane sulfonate (EMS) mutant screen. Meanwhile, we provide new insights into the influence of ethylene on flower senescence with a plausible explanation of the formation of dried flowers in *G. paniculata*. This chromosome-scale reference genome and the genetic mechanisms governing floral development in *G. paniculata* offer valuable resources for developing consumer-oriented selective breeding of *Gypsophila*.

## Results

### *G. paniculata* genome assembly and annotation

**Figure 1:**
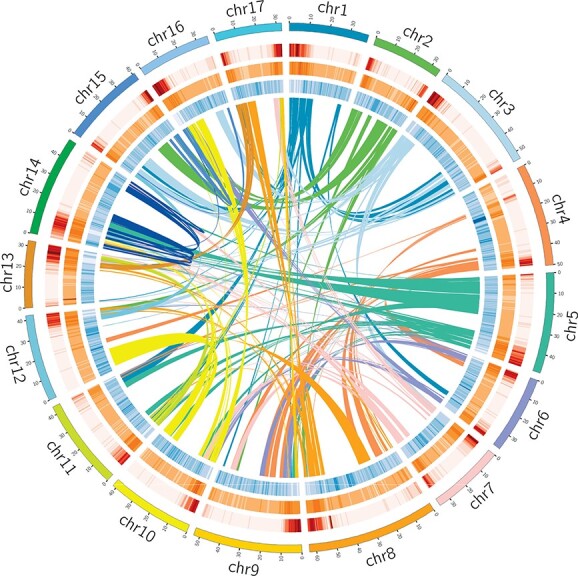
Overview of the *G. paniculata* genome assembly. **a**, The 17 pseudochromosomes illustrated in different colors, **b**, gene density (sliding window size = 500 kb), **c**, transposon elements (sliding window size = 500 kb), **d**, tandem repeats (sliding window size = 500 kb), and **e**, connections of collinear blocks (with the same colors as pseudochromosomes) of the *G. paniculata* genome.

The genome sequencing of *G. paniculata* was conducted from a wild-type plant with pink single flowers. The genome was sequenced using Illumina HiSeq, Nanopore sequencing, and Hi-C technique. Approximately 82.85 Gb of Nanopore long reads were generated, covering 102.92-fold of the 805.00 Mb genome sequence (k-mer analysis data, Figure S1, see online supplementary material). The genome size of *G. paniculata* was 794.66 Mb determined by flow cytometry (Figure S1, see online supplementary material), which was in line with the k-mer estimate (k = 17). After the pipeline of the MECAT assembling program and PILON, the long-read reads were assembled into a contig level with error sites corrected. A total of 749.58 Mb error-corrected contig-level assembly was obtained (281 contigs, N50 = 15.07 Mb, Table 1). In the end, the assembly results at the chromosome level, which is combined by 17 pseudo chromosome scaffolds and 82 contigs that are not anchored to any chromosome, were obtained after adjusting the assembly results produced by ALLHiC (Figs 1 and S2, see online supplementary material). According to the alignment result with eudicotyledons_odb10 using BUSCO, 93.8% universal single-copy orthologous gene groups in eudicot were obtained in the genome assembly, indicating that the result of the assembling process is relatively integrated. We adopted bwa-mem2 for aligning NGS (Next generation sequencing) reads to the assembly. It turned out that 99.84% of the NGS reads are mapped to the reference genome, and 96.56% are properly paired. By aligning the genome assembly with several assemblies of the mitochondrion and the chloroplast using BLASTN (version = 2.12.0+, *P*-value $\le$0.001), we confirmed that the genome assembly covered a part of the mitochondrion and most of the chloroplast (Table S1, see online supplementary material).

**Table 1:** The statistics for genome sequencing of *G. paniculate*.

Items	Number	Size	Coverage
Nanopore reads	3 574 137	82.85Gb	
Total contigs	281	749.58 Mb	
Contig N50	18	15.07 Mb	
Total protein-coding genes	24 459		
Total repetitive sequences	895 344	571.71 Mb	77.23%
LTR retrotransposon	266 136	352.90 Mb	47.67%
Non-LTR retrotransposon	93 578	36.22 Mb	4.89%
DNA transposon	265 560	132.31 Mb	17.87%
Tandem repeats	200 870	52.22 Mb	7.05%

After the pipeline of MAKER, 24 459 genes and 40 273 transcriptions were annotated. By the alignment with eudicotyledons_odb10 using BUSCO, 90.07% universal single-copy orthologous gene groups in eudicot were found in the annotation. After the adoption of a process combined by RepeateModeler and RepeatMasker, a total of 895 344 repeat sequences covering ~77.23% of the genome were obtained (571.74 Mb), consisting of retroelement (71.33%), DNA Transposon (23.14%), and tandem repeats (9.13%). Interestingly, we found a remarkable feature in the distribution of genes in the assembly of *G. paniculata* genome: the gene density is much higher at the ends of chromosomes than central (Fig. 1), which is far more different from the vast majority of dicotyledonous plants’ genomes [28]. A significant negative correlation was shown between chromosome gene distribution density and distribution of average length of introns (Figure S3, see online supplementary material). Therefore, a hypothesis is raised that the distribution of extra-long introns might reduce the distribution density of genes, which was also reflected in the human genome [29].

### Whole-genome duplication and genome evolution analysis

**Figure 2:**
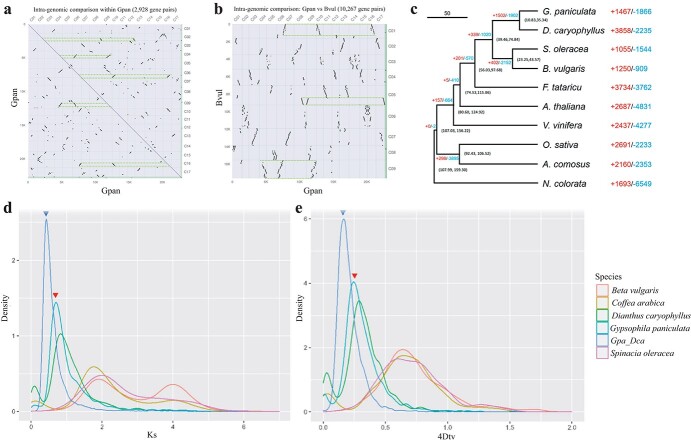
Evolution analysis of *G. paniculata* and relative species. **a**, Collinearity blocks within the genome of *G. paniculata*. **b**, Collinear blocks between the genomes of *G. paniculata* and *B. vulgaris*. The collinear blocks in (**a**) show a 1:2 relationship and these in (**b**) show a 1:3 relationship as indicated by green dotted lines, which intuitively reveals the WGT event happened between the divergence of *B. vulgaris* and *G. paniculata*. **c**, Divergence time and ortholog expansion of *G. paniculata* and close species. The unit of the 95%highest probability density confidence intervals for species divergence time is MYA (million years ago). **d** and **e**, Ks and 4Dtv distribution density of synteny genes within and between different species. Gpa_Dpa indicates the collinearity between *G. paniculata* and *D. caryophyllus*. The blue triangles indicate the divergence of *G. paniculata* and *D. caryophyllus*, while the red ones indicate the WGT event on their MRCA.

To investigate whole-genome duplication events during the evolutionary process of *Gypsophila*, we first clustered the orthologous genes derived from the intergenomic and intragenomic analysis of *G. paniculata*, *Dianthus caryophyllus*, *Spinacia oleracea*, *Beta vulgaris*, *Fagopyrum tataricum*, *Arabidopsis thaliana*, *Vitis vinifera*, *Oryza sativa*, *Ananas comosus*, and *Nymphaea colorata*. Then single-copy orthologs were adopted for the construction of the phylogenetic tree. Synonymous substitution is thought to occur at a relatively stable rate during the evolution process of orthologous genes, allowing them to be used as molecular clocks due to their absence from selection [30]. Ks and 4Dtv distribution of synteny gene pairs within collinearity blocks were used for speculating the whole-genome duplication events. Long collinearity blocks detected within *G. paniculata* (Fig. 2a) and between *G. paniculata* and *B. vulgaris* (Fig. 2b) showed the sign of a recent whole-genome triplication (WGT) event. A significant expansion of orthologs of the MRCA (most recent common ancestor) of *G. paniculata* and *D. caryophyllus* was spotted (Fig. 2b). Both distributions of 4Dtv and Ks revealed that a polyploid event had occurred before the divergence of *G. paniculata* and *D. caryophyllus* (Fig. 2c, d, e). The results indicated that *G. paniculata* shared a most recent polyploidy event with *D. caryophyllus*, which may play an essential role in the evolution of the Caryophyllaceae family. Last, the evolution analysis was investigated between the species in Caryophyllidae, Rosidae and Asteridae, with the additional genome data of *Coffea arabica*, *Corymbia citriodora*, *Cuscuta australis*, *Malus domestica*, *Pyrus communis*, and *Salvia splendens*. In order to reduce the instability during phylogenetic analysis, the longest transcription of each gene was chosen with other sequences being removed. The filtered sequences were subsequently analyzed by OrthoFinder, and the phylogenetic tree was reconstructed to reveal the evolutional relationship among the selected species. The phylogenetic tree supported that Caryophyllidae is an outgroup to both Rosidae and Asteridae, and Caryophyllidae is closer to Rosidae phylogenetically compared to Asteridae (Fig. S4, see online supplementary material).

### Genetic basis of floral development in *G. paniculata*

Through the blast of identified MADS-box genes from *Arabidopsis* in *G. paniculata* genome, 95 MADS-box genes were identified, including 33 Type I MADS-box genes and 62 Type II MADS-box genes (Fig. S5 and Table S2, see online supplementary material). In the phylogenetic tree constructed together with several other angiosperm species, the Type II MADS-box genes of *G. paniculata* are divided into 14 groups, namely *SEPALLATA* (*SEP*), *AGAMOUS-LIKE 16* (*AGL16*), *APETALA 1* (*AP1*), *FLOWERING LOCUS C* (*FLC*), *AGAMOUS*/*SEEDSTICK* (*AG*/*STK*), *SUPPRESSOR OF OVEREXPRESSION OF CO 1* (*SOC1*), *SHORT VEGETATIVE PHASE* (*SVP*), *AGAMOUS-LIKE 15* (*AGL15*), *ARABIDOPSIS NITRATE REGULATED 1* (*ANR1*), *AGAMOUS-LIKE 12* (*AGL12*), *OsMADS32*, *APETALA 3/PISTILLATA* (*AP3/PI*), *ARABIDOPSIS BSISTER* (*BS*), and MIKC^*^. *TM8* and *AGL32* were found neither in the *G. paniculata* genome nor the transcriptome. In addition, no significant expansion of gene number in the 11 groups of Type II MADS-box genes was detected in *G. paniculata*, except for *OsMADS32*, *FLC*, and *AGL12*.

The phylogenetic profiles of the genes related to flower development in *G. paniculata* were further investigated, and the homologous genes for the ABCE model were identified (Fig. S6, see online supplementary material). There are three *AP1* homologs of A-class genes and three B-class genes consisting of two *AP3* genes and one *PI* gene. Both duplicates of the C-class genes, *AGa* and *AGb*, are retained on the collinear blocks, which likely results from the genome duplication event of *G. paniculate*, as the *STK* group only consists of one member. In addition, four *SEP* homologs belonging to the E-class are found. To conclude, the distribution and number of ABCE genes in *G. paniculata* are similar to those in *Arabidopsis*, indicating that there is no significant expansion or loss of genes during the whole genome duplication of *G. paniculata*.

### Double flower regulation and the relative genes in *G. paniculata*

**Figure 3:**
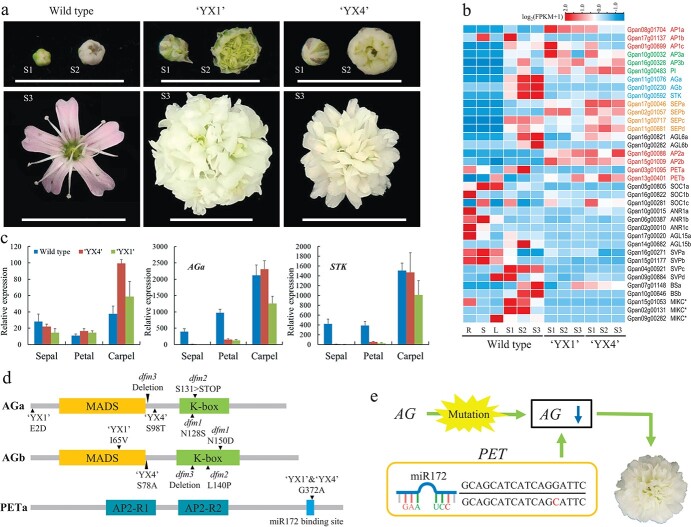
The MADS-box gene expressional profile and proposed regulation network of double flower formation in *G. paniculata*. **a**, The morphological characteristics of the single-flowered (wild type) and double-flowered (‘YX1’ and ‘YX4’) *G. paniculata* were displayed here. Three distinguishable flower development stages were shown for each variation, namely flower bud stage (S1), semi-open stage (S2), and fully open stage (S3). **b**, The transcriptome analysis of *G. paniculata* MADS-box genes from three flower development stages in various flower types. Four group genes (ABCE) were classified according to the expression of MADS-box genes. Expression values were scaled by log_2_(FPKM +1), in which FPKM is fragments per kilobase of exon per million mapped reads. **c**, qPCR-based gene expression level of *PETa, AGa*, and *STK* in different floral organs of the fully open stage (sepal, petal, and carpel). Because no stamen was found in two double flower cultivars, we did not detect gene expression in that organ. The expression of more ABCE model genes were shown in Figure S7 (see online supplementary material). **d**, The representative detected mutations in *AG* and *PET* genes from EMS mutants and cultivars (detailed mutation information is shown in Table S3, see online supplementary material). **e**, The regulation network of double flower formation in *G. paniculata*.

To investigate the organ determination and development of double flowers, the floral development process was divided into three stages in wild type and double flower cultivars (‘YX1’ and ‘YX4’), namely flower bud stage, flower semi-open stage, and flower fully open stage (Fig. 3a). ‘YX1’ and ‘YX4’ are two representative commercial varieties of *G. paniculata* with white petals, and the difference is that the flower type of ‘YX1’ is larger than that of ‘YX4’. We analysed the RNA-seq data of type II MADS-box genes and generated its expression patterns in the whole flower and different floral developmental stages (Fig. 3b). The results showed that the expression level of *AG* genes (C-class) is largely decreased in two double flower cultivars compared with wild type, while the expression levels of A-, B-, and E-class genes increased to a first approximation. We then further investigated the expression pattern of ABCE model genes in different floral organs (sepal, petal, stamen, and carpel) at the fully open stage (Fig. S7, see online supplementary material). We did not detect the gene expression in stamen as it was not found in two double flower cultivars. In total, 15 genes (*AP1a/b/c*, *AP2a/b*, *PETa/b*, *AP3a/b*, *PI*, *STK*, *AGa/b*, and *SEPa/b*) were examined in different floral organs. All the expression levels of the C-class genes were reduced significantly in petal of double flower cultivars, while the expression levels of B-class genes *PI* and *AP3b* increased dramatically in the same organs, compared with those in wild type. The relative expression levels of A-class genes were quite low in floral organs except for *AP1a* and *PETa.* Although slight differences were displayed between double flower cultivars and wild type, no clear pattern was summarized. The E-class genes *SEPa* and *SEPb* showed different expression patterns as well. *SEPa* expressed more or less in all the samples, whereas the expression levels were significantly high in floral organs of double flowers rather than wild type.

Previously, three double flower mutants of *G. paniculata* were isolated, namely *dfm1*, *dfm2*, and *dfm3*, through EMS induced mutation using single flower wild type seeds. The three double flower mutants displayed unequal variation in flower type and color compared with wild-type flowers (Fig. S8b, see online supplementary material). The *AG* genes of the three mutants were then cloned, and SNP switches and deletions were identified in the *AG* sequences (Fig. 3d and Table S3, see online supplementary material). In addition, the *AG* genes in ‘YX1’ and ‘YX4’ also showed several non-synonymous mutations, which might lead to the dysfunction or expression disorder of *AG* and cause the double flower phenotype. Besides, the miR172 binding site (GCAGCATCATCAGGATTC) of *AP2/PET* is also responsible for the double flower formation in flower plants [9]. An SNP in *PETa* miR172 binding site (G to C) was detected in ‘YX1’ and ‘YX4’, but not in *dfm* lines. Lastly, random crosses between wild type and commercial double flower cultivars were performed, and several double flower progenies were isolated in the seeds harvested from wild type, indicating the dominance of the double flower phenotype (Fig. S8c, see online supplementary material). Similar to the known theory [9], our data suggest that *AG* and *PET* genes are the key genes in the regulation of double flower formation in *G. paniculata* in this research (Fig. 3e).

### Flower senescence and ethylene response in *G. paniculata*

**Figure 4:**
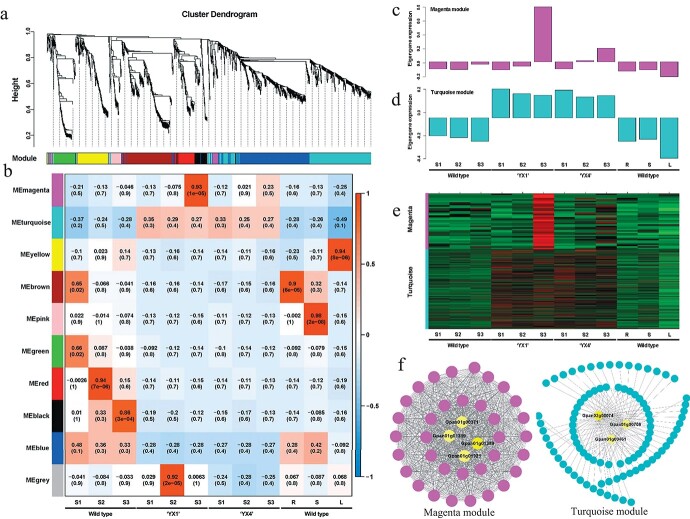
WGCNA of expressed genes among different tissues in wild type, ‘YX1’, and ‘YX4’ cultivars. **a**, Hierarchical cluster tree showing co-expression modules identified by WGCNA. Each leaf in the tree is one gene. The major tree branches constitute 10 modules labelled in different colors. **b**, Heatmap showing cluster-tissue associations. Each row corresponds to a cluster. Each column corresponds to a specific tissue. The color of each cell at the row-column intersection indicates the eigengene expression value. The blue color indicates a negative association and the red color indicates a positive association between the cluster and the tissue. Specific cluster eigengene values are shown in the figures. **c** and **d**, Eigengene expression profile for the magenta and turquoise module in different samples. **e**, Heatmap showing the relative FPKM of each gene from the magenta and turquoise module. **f**, The gene network of magenta and turquoise module. The edge weight ranked top 150 is visualized by Cytoscape. The hub candidate genes are marked with yellow.

RNA-seq data of different samples were generated from *G. paniculata* wild type, ‘YX1’ and ‘YX4’ in the exploration of mechanisms underlying the trait of *G. paniculata* befitting for dried flowers. A total of 23 449 expressed genes were clustered into 10 different color modules using WGCNA package on the basis of pairwise correlations between genes in their common expression trends among all samples. The 10 different color modules are shown by the dendrogram in Fig. 4a, in which each tree branch constitutes a module and each leaf in the branch is one gene. Notably, the magenta and turquoise modules are associated with the floral development stages in the sample of two cultivars, suggesting the genes in those modules might be involved in the flower development and senescence (Fig. 4b).

In the magenta module, 406 genes are highly expressed at the flower fully open stage (S3) in two cultivars. Based on the gene expression pattern and annotation, we obtained four genes that might be associated with flower development and senescence (*Gpan01g00371*, *Gpan01g01321*, *Gpan01g01319,* and *Gpan01g01389*, Figure S9 and Table S4, see online supplementary material). *Gpan01g00371* and *Gpan01g01321*, the *SENESCENCE-RELATED GENE 1* (*SRG1*) like genes, are highly expressed at the S3 stage in ‘YX1’ compared with other two plants, suggesting that the senescent process is accelerated in cultivar ‘YX1’ compared with wild type and ‘YX4’. Likely, *Gpan01g01319* encodes a purple acid phosphatase, which regulates phosphate during leaf senescence and is upregulated during leaf senescence [31]. The expression of *Gpan01g01319* is all upregulated during flower development in wild type and two cultivars, indicating that it is possibly involved in petal senescence as well. Moreover, *Gpan01g00371, Gpan01g01321,* and *Gpan01g01319* also show hub roles in the gene networks in the magenta module, together with the unknown gene *Gpan01g01389* (Fig. 4f). Different to the senescence marker genes, two genes that potentially delay plant senescence, *Gpan07g00598* (gibberellin receptor) and *Gpan05g00038* (peroxidase 5, an antioxidant enzyme), are highly expressed at stage S3 in wild type rather than that in the two cultivars. The accumulation of peroxidase helps to eliminate reactive oxygen species in plants and thus delays senescence [32]. Our data collectively imply that the above genes possibly regulated the flower senescence. Thus, it is possible that the *G. paniculata* naturally harboured the ability of anti-senescence and this ability was partially lost during the process of artificial breeding.

There are also 5560 co-expressed genes clustered into the turquoise module, associated with different floral development stages (S1 to S3) in the two cultivars. A lot of genes related to ethylene signal transduction and response were discovered and clustered in the turquoise module (Fig. S9 and Table S5, see online supplementary material), including *ETHYLENE RESPONSE SENSOR* (*ERS*, *Gpan17g00803*, *Gpan15g00618*, and *Gpan13g00217*), *ETHYLENE INSENSITIVE 3-LIKE* (*EIL*, *Gpan04g00597*), and *ETHYLENE-RESPONSIVE TRANSCRIPTION FACTOR* (*Gpan02g00593* and *Gpan08g01162*). Most of the ethylene-related genes show lower expression levels in “YX1” and “YX4”, suggesting that the two cultivars might be insensitive to the ethylene, resulting in the delayed flower senescence. Three unknown genes, *Gpan02g00074*, *Gpan01g00708,* and *Gpan01g00461*, show the hub regulation roles in the gene network of the turquoise module, indicating that they may have functions in the regulation of flower senescence in the two cultivars (Figure 4f). The RNA-seq data provides some candidate genes that might be involved in the flower senescence, and also suggests that the delay of flower senescence might be determined by the ethylene insensitivity of *G. paniculata*.

**Figure 5:**
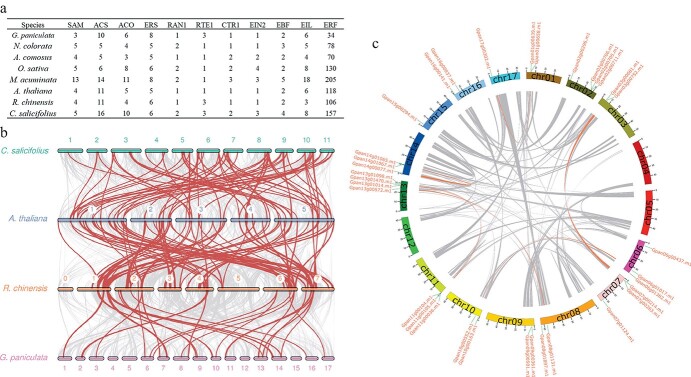
The key genes of *G. paniculata* involved in ethylene biosynthesis and signaling transduction pathways. **a**, The number of key genes regulating ethylene biosynthesis and signaling transduction. The species were selected from basal angiosperm *N. colorata,* monocots (*A. comosus, O. sativa,* and *M. acuminata),* and eudicots (*A. thaliana, R. chinensis, C. salicifolius,* and *G. paniculata*). **b**, Synteny analysis of *ERF* genes between *G. paniculata, C. salicifolius, A. thaliana,* and *R. chinensis.* Gray lines in the background indicate the collinear blocks within *G. paniculata* and other plant genomes, while the red lines highlight the syntenic *ERF* blocks. **c**, Schematic representation for the chromosomal distribution and interchromosomal relationships of *G. paniculata ERF* genes.

Next, the key genes involved in the pathways of ethylene biosynthesis and signaling transduction were investigated through genomic data of eight species, including early angiosperm (*Nyctophila colorata*)*,* monocots (*Ananas comosus, O. sativa,* and *Musa acuminata)*, and eudicots (*A. thaliana, Rosa chinensis, Chimonanthus salicifolius* and *G. paniculata*). Eleven core gene families were identified via comparative genome analysis, namely *S-ADENOSYL METHIONINE (SAM)*, *1-AMINOCYCLOPROPANE-1-CARBOXYLIC ACID SYNTHASE* (*ACS*), *1-AMINOCYCLOPROPANE-1-CARBOXYLIC ACID OXIDASE* (*ACO*), *ERS*, *RESPONSIVE TO ANTAGONIST 1* (*RAN1*), *REVERSION TO ETHYLENE SENSITIVITY 1* (*RTE1*), *CONSTITUTIVE TRIPLE RESPONSE 1* (*CTR1*), *ETHYLENE INSENSITIVE* (*EIN*), *EIN3-BINDING F-BOX PROTEIN* (*EBF*), *EIL*, and *ETHYLENE RESPONSE FACTOR* (*ERF*) (Fig. 5a). Among these gene families, only one member is detected in *RAN1*, *CTR1*, and *EIN* gene families in *G. paniculata*, and the gene numbers of other families in *G. paniculata* genome are close to that of other monocots and eudicots, like *SAM*, *ACS*, *ACO*, and *RTE1* genes. Interestingly, the *ERS* genes, located on the membrane and transporting ethylene signal from extracellular to the cytoplasm [33], have the largest gene number (eight) in *G. paniculata*, similar to in banana (*M. acuminata*). A huge gene number shrinkage is identified in *ERF* family, in which only 34 genes are detected in *G. paniculata*, while there are 205 and 118 members identified in banana and *Arabidopsis*, respectively, implying that there is a dramatical reduction of the *ERF* gene number in *G. paniculata*.

A comparative syntenic map was further constructed using *C. salicifolius, A. thaliana, R. chinensis,* and *G. paniculata* to infer the narrowed mechanism of the *ERF* gene family in *G. paniculata* (Fig. 5b and c). In the genome of *G. paniculata*, all of 34 *ERF* genes are mapped onto 14 chromosomes, except the chromosomes 4, 5, and 12. In detail, four *ERF* genes are located on chromosome 2 and 13, while only one member is found on chromosome 15 and 17, respectively. Moreover, six duplicated syntenic blocks composed of 22 *ERF* genes are identified on ten chromosomes of *G. paniculata* genome, all generated from segmental duplication. Besides, six tandem duplication *ERF* gene pairs are also found on chromosomes 1, 2, 7, 11, 13, and 14*.* However, a total of 71 interspecific syntenic blocks are identified between *C. salicifolius* and *A. thaliana,* and 76 interspecific syntenic orthologous gene pairs are found in *A. thaliana* and *R. chinensis,* whereas only 25 syntenic blocks exist between *R. chinensis* and *G. paniculata.*

**Figure 6:**
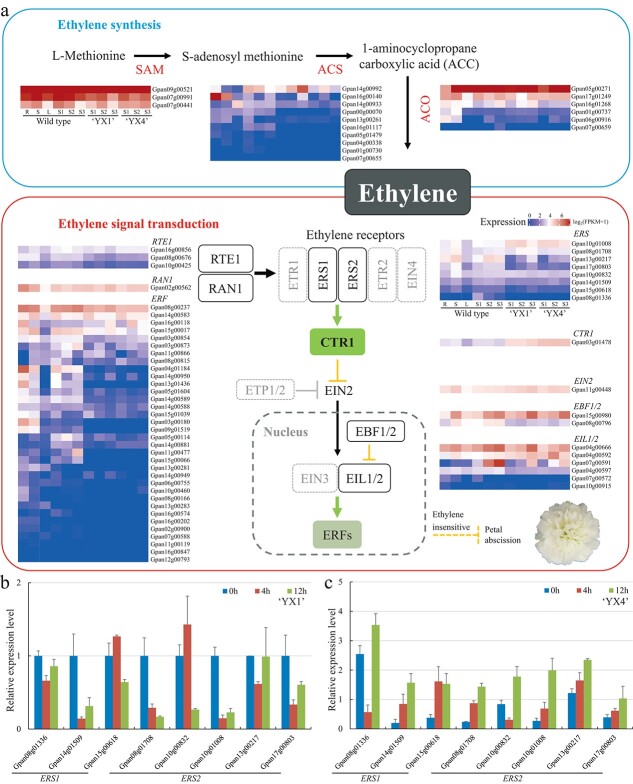
The ethylene biosynthesis and signaling transduction pathways of *G. paniculata*. Heat maps of the expression of ethylene biosynthesis and signaling transduction pathway genes during flower development in wild type and two cultivars (‘YX1’ and ‘YX4’). The R, S, and L refer to root, stem, and leaf. The S1, S2, and S3 are flower bud stage (S1), semi-open stage (S2), and fully open stage (S3), respectively. **b** and **c**, Expression of *ERS* genes in petals of two cut *G. paniculata* flowers determined by qPCR. Flowers were treated with 10 ppm ethylene for 0 h, 4 h, and 12 h. The air treatment (0 h) was used as a control. ‘YX1’ and ‘YX4’ were the name of two cultivars used in the experiment.

The expression profiles of the above gene families in different flower development stages and tissues were conducted using *G. paniculata* wild type and two cultivars (Fig. 6). The ethylene synthesis and signal transduction genes are highly expressed in all tissues and development stages, such as *SAM*, ACO, *RAN1*, *EIN2*, *EBF*, and *EIL*. However, the expression level of *ERS* is relatively low in all detected tissues, indicating the *ERS* genes were not highly active during the floral development of *G. paniculata*. As expected, *ERFs* show much lower expression during plant development, except for one gene (*Gpan03g00752*).

To support the hypothesis that the insensitivity of *G. paniculata* to ethylene is due to the abnormal function of ethylene receptors, we investigated the expression level of *ERS* under short exposure (0 h, 4 h, and 12 h) to exogenous ethylene using the double flowers of two cultivars (‘YX1’ and ‘YX4’). Eight *ETR* genes identified in the genome of *G. paniculata* were detected, which could be divided into two groups based on the phylogenetic analysis, namely *ERS1* and *ERS2* (Fig. 6b and c). The expression of the *ERS1* and *ERS2* genes decreased significantly after ethylene treatment in cultivar ‘YX1’, while two *ERS1* and *ERS2* genes (*Gpan08g01336* and *Gpan10g00832*) showed decreasing after 4 h ethylene treatment in cultivar ‘YX4’. This is also consistent with the observed senescent process that ‘YX1’ ages faster than ‘YX4’. These results prove that ethylene receptors of *G. paniculata* do not respond to exogenous ethylene in commercial cultivars and the ethylene sensitivity varies in different varieties, which may be caused by artificial selection during plant breeding. Our data collectively suggest that the low expression level of *ERS* genes and the reduced number of downstream *ERF* genes might be related to ethylene insensitivity in *G. paniculata* due to non-ethylene response, resulting in the petal reservation during the process of flower senescence.

## Discussion

*G. paniculata*, the only species used as cut flowers in the genus *Gypsophila*, is an important cut flower for global floricultural markets. In this study, we have sequenced the genome of *G. paniculata* wild type, which is the first genome of the *Gypsophila* genus. On the premise of a pseudochromosome genome assembly and a relatively complete annotation, the WGT events are well illustrated in different ways, such as genome collinearity and orthologs analysis, which are shared by *G. paniculata* and *D. caryphyllus* and may play an essential role in the evolution of *Caryophyllaceae.*

The genome of *G. paniculata* provides a better understanding of floral development, which is the key ornamental trait in its breeding process [34]. The application of databases about the floral development regulation networks in several flowering plants serves to identify the homologs of floral development genes in *G. paniculata*, together with the comparative transcriptome data analyses. In the expression profile of MADS-box genes in *G. paniculata*, the expression of *AG* genes showed a significant decrease in double flower cultivars, whereas the expression of *AP2*/*PET* genes showed a sharp increase in the whole flower at different stages. In EMS mutants and double flower cultivars, variety mutations were identified in *AG* genes, which is a core gene playing an essential role in double flower formation in many flowering plants [35–38]. For instance, *AGa* and *AGb*, the *AG* subfamily, are widely expressed in stamens and carpels in most flower plants, such as *Nymphaea colorata*, *D. caryphyllus*, and *Eustoma grandiflorum*, and the mutation or reduced expression in petal of *AG* will cause the formation of double flower [39–41]. Moreover, it has been reported that mutations in the miR172 target site of *AP2*/*PET* were responsible for the double flower formation in *Arabidopsis*, petunia, rose, peach, and *Dianthus* [42]. In present study, we detected an SNP substitution in *PETa* from two double flower cultivars, but no mutations in EMS mutants, which had fewer petals compared to the cultivar. This could be that the disrupted *AG* have a cumulative effect with overaccumulation of PET on double flower formation. In all, the revealing of the floral development regulation in *G. paniculata* provides the genetic basis for molecular breeding using genetic engineering technology, such as genome editing.

Dried flowers and dyed flowers are important forms of cut *G. paniculata* flowers. Our results suggested that low expression of ethylene receptors and the massive loss of ethylene response factors were related to ethylene insensitivity during flower senescence. This phenomenon has also been observed in carnation flowers, which are highly sensitive to ethylene but petals do not fall naturally [42, 43]. Six putative ethylene receptors were identified in carnation genomic sequences, and *ETR1*, *ERS1,* and *ERS2* genes were classified in one subfamily via phylogenetic analysis. These three genes were expressed constitutively or at undetectable levels in different floral tissues during flower senescence in carnation reported by previous independent research [44]. We found the ethylene response factors, *ERF* genes, were largely lost in *G. paniculata* genome (34 members in *G. paniculata*, while there were 205 and 118 members identified in banana and *Arabidopsis*), as well as in carnation genome (41 *ERF*s of carnation vs 106 *ERF*s of rose) [45]. These results were in line with our data in *G. paniculata* during flower senescence. Moreover, similar results have also been achieved in geranium and *Delphinium*, demonstrating that the effect of ethylene on flower senescence may not be carried out by regulating the expression of ethylene receptor genes [46–48]. Thus, we speculate that the partial gene loss and low ethylene response of ethylene receptors during the evolution of Caryophyllaceae species changed the ethylene response network, which basically abandoned the gene function of ethylene response factors, resulting in the low expression level and massive gene loss of *ERF* genes.

## Conclusions

In summary, we have presented a high-quality genome of *G. paniculata*, the first reference genome of the *Gypsophila* genus. This *G. paniculata* genome will be a significant contribution to this ornamental plant research community. The whole-genome duplication signatures demonstrate two major duplication events in the evolutionary history of *G. paniculata*. Through this high-quality chromosome-level genome, we identified and analysed the MADS-box genes to illustrate the floral development regulation of *G. paniculata*. Notably, with the sequence polymorphism analyses of EMS double-flowered mutants and commercial cultivars, the disruption of *AG* and/or the misregulation of miR172-mediated *PET* expression are found to be associated with the double flower formation. We also found that the transcriptional regulation and the reduction in the number of ethylene-related genes lead to the insensitivity of *G. paniculata* to ethylene, satisfying the characteristics of dried flowers. This *G. paniculata* genome will also serve as a great resource to improve ornamental traits through molecular breeding in the future due to the fact that *G. paniculata* is increasingly consumed and demanded for new varieties in the global floricultural market.

## Materials and methods

### Genome and transcriptome sequencing

Total DNA for genome sequencing was extracted from fresh young leaves of a single wild type plant of *G. paniculata*. RNA for transcriptome sequencing was extracted from several organs and tissues of wild type, ‘YX-1’ and ‘YX-4’, including root, shoot, leaf, flower bud, semi-open flower, and fully open flower.

After the samples were qualified, the genomic DNA was randomly cut off with Megaruptor. Large DNA fragments were enriched and purified by magnetic beads. BluePippin automatic nucleic acid recycling instrument is used to cut and recover large fragments and repair the damage of fragmented DNA. After purification, the terminal repair was carried out at both ends of the DNA fragment. After purification, the joints in the LSK108 connection kit were used for connection reaction. The Qiagen kit was used to extract high-quality DNA, and a Nanopore library with insertion fragments larger than 20 KB was constructed. The Oxford Nanopore Technology (San Diego, California, USA) sequencer was applied to conduct single-molecule real-time sequencing of DNA, after which the original reads were filtered to remove low-quality data and adapter sequences.

### Estimation of genome size

The Jellyfish (https://github.com/gmarcais/Jellyfish) program was used for K-mer (k = 17) distribution analysis of *G. paniculata* to estimate the genome size. For the low error rate of next-generation sequencing, it is commonly used in K-mer distribution analysis, whose correctness is closely related to the accuracy of the sequencing result. At the same time, we also performed the genome size determination by a flow cytometer (BD FACScalibur, San Diego, California, USA,, BD Bioscience, USA) using leaf samples. The nuclei of the samples were stained with DNA fluorochrome PI (propidium iodide, 50 mg/mL) and were measured through flow cytometer with at least 10 000 nuclei. The coefficient of variation (CV) of both sample and reference for every measurement was controlled within 5%. The genome size was determined by comparing with a standard reference genome of *Oryza sativa L. japonica*. cv. Nipponbare (genome size is 420 Mb) based on the method of Arumuganathan and Earle [49].

### Genome assembly

We applied MECAT process [50] to assemble the fragments in the constructed library to the contig level. High-quality read data from the next-generation sequencing were used to correct low-quality assembly results of the nanopore sequencing by pilon (https://github.com/broadinstitute/pilon). The Hi-C sequencing result was adopted for the construction of pseudo chromosome level scaffolds. BWA (0.7.17-r1188) [51] was applied for the alignment of Hi-C reads against contig sequences, then low-quality alignments were removed. The optimized alignment result is used by ALLHiC (v0.8.11) process [52] for preliminary assembly. In the end, the assembly results at the chromosome level were obtained after adjusting the assembly results produced by ALLHiC.

We aligned the NGS reads to the assembly using bwa-mem2 (v2.2.1) (bwa-mem2 mem -t 40 -o Gpan.sam Gpan_WG.chromosome.fasta.gz R1.fastq.gz R2.fastq.gz) [53]. Samtools (v1.15.1) was chosen to calculate the mapping rate of NGS reads in the sam file (samtools flagstat Gpan.sam) [54]. BUSCO (Benchmarking Universal Single-Copy Orthologs) [55] is a widely adopted method to evaluate the integrity of genome assembly and annotation. We used BUSCO (v3.1.0, python run_BUSCO.py -i Gpan.asm.fasta -o Gpan.genome.busco -l ~/data/busco/eudicotyledons_odb10/ -m genome -c 1) to align the assembly result with the database named eudicotyledons_odb10 to find that 93.8% of universal single-copy orthologous gene groups in eudicot were aligned with it.

### Genome annotation

The protein sequences of *S. oleracea*, *B. vulgaris*, *F. tataricum*, *A. thaliana*, *V. vinifera*, *O. sativa*, *A. comosus*, and *Nymphaea colorata* were used as homologous reference sequences to predict the gene structure in the genome. Combined with the transcriptome data, the maker (http://www.yandell-lab.org/software/maker.html) was applied to annotate the genome. After the structural annotation, we applied BUSCO to judge the quality of the annotation (python run_BUSCO.py -i Gpan.pep -o Gpan.prot.busco -l ~/data/busco/eudicotyledons_odb10/ -m proteins -c 1). Then we used eggnog-mapper [56] (emapper.py -i Gpan.pep -d virNOG -o Gpan_genes.NOG.virNOG —cpu 20 -m diamond) and HMMsearch [57] (hmmscan -o out.txt —tblout out.tbl —cpu 20 —noali -E 1e-5 ~/data/pfam/Pfam-A.hmm Gpan.pep) against Pfam database (http://pfam.xfam.org) to annotate the genome functionally. MCScan, a collinearity-analysing tool contained in JCVI toolset [58], was applied to analyse the collinearity of genomic genes (python -m jcvi.compara.catalog ortholog —dbtype = prot —no_strip_names Gpan Gpan). Using the circos software, we drew a circos diagram to describe the basic information of the genome including chromosome length, gene density, transcription element density, and genome collinearity.

For repetitive sequence annotation, both de novo- and homology-based approaches were applied for more comprehensive results. RepeatMasker (RepeatMasker -gff -pa 20 -lib consensi.fa.classified Gpan.genome.fasta) and RepeatModler (RepeatModeler -pa 20 -engine wublast -database dbname) were used for the searching and detection of transposable elements and Tandem Repeat Finder for tandem repeat sequences.

### Gene family analyses and species phylogenetic tree

To study the evolutional status of *G. paniculata*, we analysed the gene families with the genome of *G. paniculata*. The protein sequences of *G. paniculata*, *N. colorata colorata*, *Amborella trichopoda*, *V. vinifera*, *Solanum lycopersicum*, *A. thaliana*, *O. sativa*, *A. comosus,* and *Sorghum bicolor* were selected to construct a species phylogenetic tree. We clustered the datasets of proteins of chosen species into orthologs and paralogs at first, then filtered the genes of single-copy orthologs for the alignment and construction of species phylogenetic tree. We chose Orthofinder, a commonly adopted gene-family-clustering tool, as the main tool of the following analysis (orthofinder -t 20 -a 20 -f July11 -S diamond -A mafft). It is an integrated workflow combined with mainstream bioinformatic tools, such as DIAMOND and IQTREE, which can separate and cluster protein sequences from different genomes into paralogous gene groups and orthologous gene groups.

### Estimation of divergence time and gene family expansion

Before further analysis, we get the calibrated divergence time of *B. vulgaris* and *S. oleracea* and of *O. sativa* and *A. comosus* from the TimeTree database, whose divergence records are usually supported by synthetical research. The calibration time is then used as a correction to our analysis in further construction of divergence time. To estimate the divergence time, single-copy orthologous genes are in need to analyse the fourfold degenerate site. After the single-copy ortholog orthologous genes and basic phylogenetic tree were obtained, the longest coding sequences (CDS) of each single-copy orthologous gene was extracted according to the orthofinder results. Clustalo (v1.2.4) [59] was used for multiple sequence alignment of CDSs, and the results after gap removal were set to phylip format. We adopted MCMCtree, a tool comprised in paml software package [60], to construct the ultrametric tree, which not only contains the phylogenetic relationship, but also contains a 95% confidence interval for the divergence time. CAFÉ (v4.2.1) [61] workflow was selected to analyse gene family expansion.

### Polyploidization events

The longest protein sequences of each gene within the genome of *G. paniculata*, *B. vulgaris*, *C. arabica,* and *D. caryophyllus* were used for synteny and collinearity detection within themselves to shed light on polyploidization events. PAML was chosen for the computing of Ks value between gene pairs of synteny gene blocks.

### MADS-box gene family analysis

We used the peptide sequences of *G. paniculata*, *N. colorata*, *A. trichopoda*, *V. vinifera*, *S. lycopersicum*, *A. thaliana*, *O. sativa*, *A. comosus,* and *S. bicolor* to analyse the differences of MADS-box genes between them. HMMsearch [57] was applied for the annotation of gene family by selecting homologous protein sequences with E value less than 1e^−8^. The abnormal sequences that are too long or too short were manually deleted, and the longest transcript was chosen when multiple transcripts coexist. Different groups of MADS-box genes were separated to be aligned by MAFFT [62] and MUSCLE [63] to construct phylogenetic trees for each gene family.

### WGCNA analysis

In order to search the hub genes involved in the flower senescence of *G. paniculata*, 21, 737 genes (FPKM ≥1, and the means variation of FPKM >0.5) were selected to do weight correlation network analysis (WGCA) using R package [64]. An adjacency matrix was constructed with power = 7 by raising the co-expression measure. Then the genes were hierarchically clustered and formed an adjacency matrix. The topological overlap (TO) was calculated by using that adjacency matrix. Hierarchical clustering tree was constructed by dynamic hybrid tree cut algorithm with 0.5 merged degree and the expression of each module gene was also generated with scaled gene expression profiles using the first principal component. The correlation of each gene module with the different samples was also qualified.

### Gene cloning and RT-qPCR analysis

The genomic and cDNA sequence of *AG*, *AP2*, and *PET* genes were isolated from the genome dataset of *G. paniculata*. The primers for gene cloning were designed by Primer Premier 5 (Table S6, see online supplementary material). PCR cloning was performed as described previously [65].

Severn *ETR* genes were identified from the genome data and used for the expression analysis RT-qPCR was performed to analyse the expression profile of the *G. paniculata*, which was treated with 10 ppm ethylene in an airtight vacuum dryer for 0 h, 4 h, and 12 h. RT-qPCR was performed using LightCycler® 480 II Real-time PCR Instrument (Roche, Basel, Switzerland) with QuantiFast® SYBR® Green PCR Kit (Qiagen, Dusseldorf, Germany). RT-qPCR reactions were performed as described previously [65]. *ACTIN* was used as a reference gene for RT-qPCR reactions. The gene-specific primers for qPCR of the target genes were shown in Table S6, see online supplementary material.

## Acknowledgements

F.L. acknowledges funding from National Natural Science Foundation of China (31960608), Yunnan Fundamental Research Projects (202101AT070147), and High-level Talent Introduction Program of Yunnan Province – Industrial Talent Special Project (YNQR-CYRC-2020-004). C.Y. thanks the Green Food Brand—Build a Special Project (Floriculture) supported by Science and Technology (530000210000000013742). J.W. is funded by China Agriculture Research System of MOF and MARA (CARS-23-G56). L.Z. thanks the Fundamental Research Funds for the Central Universities (2021QNA6008). We want to thank the support from Yuxi Yunxing Biological Technology Co., Ltd. (Yunnan, China).

## Author contributions

F.L. managed the project. F.L, L.Z., and J.W. conceived and designed the study. F.L., Y.G., C.J., and X.W. wrote the manuscript. F.L., J.R., C.Y., and J.W. collected the plant materials for sequencing and did the cross breeding. F.L., Y.C., and H.G. conducted the EMS mutagenesis experiment, cloned the genes, and analysed the sequence. Y.G., H.Q., and L.Z. assembled and annotated the genome, performed gene family clustering, and comparative phylogenomics. F.L., C.J., X.W., F.Z., and L.Z. conducted transcriptome sequencing and analysis. F.L., C.J., X.W., X.L., S.F., and F.Z. conducted the ethylene analysis. All authors reviewed and approved the manuscript.

## Data availability

All data are publicly available in the China National GeneBank (https://www.cngb.org/) under project number CNP0003304. The genome assembly sequences and gene annotations are available at https://ftp.cngb.org/pub/CNSA/data4/CNP0003304/CNS0579838/CNA0050895/.

## Conflict of interests

The authors declare no competing interests.

## Supplementary data

Supplementary data is available at *Horticulture Research * online.

## Supplementary Material

Web_Material_uhac176
